# Suicidal Thoughts and Behaviors in American Indian and Alaska Native Adolescents

**DOI:** 10.1007/s10900-024-01411-z

**Published:** 2024-10-15

**Authors:** James H. Price, Jagdish Khubchandani

**Affiliations:** 1 University of Toledo, 43606 Toledo, OH USA; 2 New Mexico State University, Las Cruces, NM-88003 USA

**Keywords:** Suicide, Adolescents, American Indian, Alaska Native, Risk Factors

## Abstract

American Indians and Alaska Natives (AI/AN) have consistently exhibited suicide rates that surpass all other racial and ethnic groups in the United States. However, not much has been published regarding the epidemiology of AI/AN youth suicides. The objectives of this study on AI/AN adolescents were to assess the prevalence of suicidal thoughts and behaviors by age and gender, assess the number of years of life lost to suicide before the age of 80, and assess the most common methods used to commit suicide by AI/AN adolescents. Data utilized for this study were from the national Youth Risk Behavior Surveys and the Web-Based Injury Statistics Query and Reporting System. We conducted a cross-sectional descriptive analysis of the suicide-related data from years 2015, 2017, 2019, and 2021. We found AI/AN adolescents averaged one in four contemplated suicides, one in five planned suicides, and one in six attempted suicides. A total of 257 adolescents committed suicide during the study period where the majority (62.5%) were observed in males and suicides were more common in older adolescents. AI/AN adolescents had the highest crude suicide death rate of any racial or ethnic group and the most common methods used to commit suicide were suffocation (57.5%) and firearms (35.5%). AI/ AN adolescents lost almost 16,000 years of potential life during the four years of the study and the majority were lost by males. Professionals and policymakers desiring to reduce suicidal thoughts and behaviors among AI/AN adolescents need to focus more of their efforts on providing youths with resilience factors to establish sufficient ego strength in them to deal with all types of stressors. Concurrently, federal, state, and tribal leaders need to work together to improve the social and economic circumstances faced by many AI/AN families and children.

Suicide is a growing public health epidemic in the United States (U.S.). The rates of suicide-related deaths prominently vary by race/ethnicity, age, gender, and other sociodemographic characteristics [1–4]. For example, compared to the national average, American Indians/Alaska Natives (AI/AN) have the highest rates of suicide surpassing all other racial and ethnic groups [2]. Especially noteworthy are the differences in suicide mortality among high school-age (14–18 years) adolescents by race and ethnicity. In 2021, AI/AN adolescents had a crude suicide rate (18.95/100,000) that was 170.4% of non-Hispanic white adolescents (11.12/100,000), 239% of non-Hispanic Black adolescents (7.93/100,000), 284.1% of Asian adolescents (6.67/100,000) and 298% of Hispanic adolescents (6.36/100,000) [3]. Suicide is the second leading cause of death for high school-age adolescents in general [1, 4]. Suicide is also the second leading cause of death for high school-age AI/AN adolescents and responsible for one-third of all the deaths in this group [4].

Suicidal behaviors and suicide have a multifactorial etiology, making the ability to predict suicide attempts extremely difficult. Suicidal thoughts (e.g. ideation and planning) and behaviors (e.g. attempts and deaths) increase with age for adolescents and their frequencies vary by gender (e.g. ideation more frequent in females versus suicide deaths more common among males) [5, 6]. Figure 1 is a conceptual diagrammatic representation of the alternative pathways that can result in adolescents moving from suicidal thoughts to behaviors. The figure is in part conceptualized from the General Strain Theory (GST) which proposes that negative life events (e.g. stressors or risk factors) can lead to negative emotional responses which lead to coping behaviors that can result in either positive outcomes (e.g. improved coping skills) or negative outcomes (e.g. substance abuse and suicidal behaviors) [7]. The Adolescent Supplement of the National Comorbidity Survey found that the lifetime prevalence of suicidal ideation, suicidal planning, and suicide attempts for adolescents (13–18 years) were 12.1%, 4%, and 4.1%, respectively [5]. The study also found that a third of those with suicidal ideation went on to develop a plan and 61% of them went on to attempt suicide. In other words, most adolescents who think about suicide do not attempt it. Of those who transition from ideation to attempt, 86% did so within a year of the onset of their ideation. Other studies of adolescents related to suicidal thoughts and behaviors found suicidal ideation/planning reached 20% and suicide attempts reached 9% in a sample living near reservations, and another study found that suicidal ideation/planning varied by gender and place of residence (reservation vs. urban) resulting in suicidal ideation/planning of 12–18% and suicide attempts from 16 to 20% [8, 9]. As illustrated in Fig. 1, there is a small portion of susceptible adolescents (possibly, as much as 30%) who are impulsive and with inadequate coping skills who move from suicidal ideation to suicide attempts within 10 min or less [10, 11]. Adolescents are often confronted with stressors that may be perceived by some as a crisis and attempt to deal with their “crises” in a state of “cognitive constriction” where they do not comprehend the impact of all of their actions and the potential consequences of the options they pursue [12]. This is not surprising since complete development of the brain does not occur until youths reach the age range of early to mid-20s [13, 14]. One of the last areas of the brain to mature is the prefrontal cortex, an area responsible for executive function (e.g. impulse control, reasoning, and decision-making) [15, 16].

**Fig. 1:**
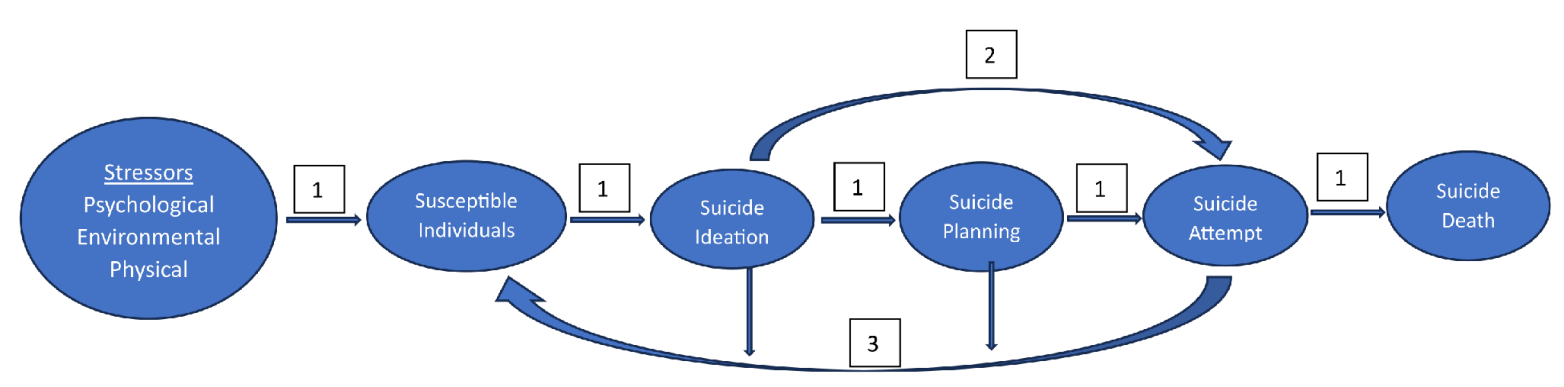
Pathways to adolescent suicidal thoughts and behaviors. 1. Traditional suicide pathway. 2. Impulsive extremely short timeframe. 3. Those who do not progress to a more dangerous stage or who survive an attempt

As previously noted, adolescents have a smaller number of suicide-related deaths as compared to the prevalence of suicidal ideation among this age group. Reasons for this disconnect include the following: an adolescent suicide attempts-related study found that 51.5% of them engaged in suicide attempts to get “relief from a terrible state of mind”, about a third simply wanted to “escape for a while from an impossible situation”, and 26.3% who attempted suicide claimed they truly wanted to die [16]. In contrast, other research studies have found an average of 50% of adolescents who attempted suicide wanted to die [17, 18]. Also, the number of suicidal deaths is related to the methods utilized by adolescents for attempting suicide. A midwestern study of youths ages 10–24 years old who attempted suicide found that two-thirds (69.1%) of those who died did so during their initial attempt [19]. Additionally, 80% were males and 85% of the initial suicidal deaths were due to the use of firearms. Males use firearms to attempt suicide more often than females. Firearms are the most lethal (90%) method utilized in a completed suicide followed by hanging/suffocation (85%), drowning (80%), falling from heights (47%), and other methods [19]. Other factors that affect the number of adolescent suicides include the availability of various methods of community suicide, the support systems for adolescents, and access to mental healthcare in communities and schools.

These findings are based on studies from adolescents across the spectrum including all races and ethnicities. Little is known about the trends and patterns of suicidal ideation, attempts, and deaths among AI/AN adolescents. Thus, the purpose of this study was to provide greater insights into the prevalence of suicides among AI/AN adolescents. Specifically, we explored the association between suicidal thoughts and behaviors among AI/AN adolescents, assessed the proportion of AI/AN adolescents who attempted suicide by age and gender, assessed the number of years of life lost to suicides among AI/AN adolescents and investigated the most common methods used for suicide among AI/AN adolescents.

## Methods

For this research, we utilized the Youth Risk Behavior Surveys (YRBS) for years 2015, 2017, 2019, 2021 and data for the same years from the Web-Based Injury Statistics Query and Reporting System (WISQARS), both datasets are publicly available on the U.S. Centers for Disease Control and Prevention (CDC) website [3, 20–23]. The YRBS surveys of 9th -12th grade students were administered in public and private schools biennially to a nationally representative sample of adolescents across 50 states and DC. Participation was voluntary, anonymous, and comprised taking a survey with 99 questions (including demographic and health risk behavior-related topics). School participation rates for the four years of the data we utilized ranged from 69 to 75% with student response rates ranging from 79 to 86% [20–23]. The specific suicidal thoughts and behaviors questions of the YRBS included the following: “During the past 12 months did you ever…” “seriously consider attempting suicide?” and “make a plan about how you would attempt suicide”. Students who answered “yes” were considered to have suicidal ideation or plans, respectively. In addition, those who claimed, “during the past 12 months, how many times did you actually attempt suicide?” and/or “made a suicide attempt that resulted in an injury, poisoning, or overdose that had to be treated by a doctor or nurse?” and responded any number of times more than zero were labeled as having a confirmed suicide attempt. We also conducted a secondary analysis of the CDC WISQARS database for repeated cross-sectional suicide mortality data (years 2015, 2017, 2019, 2021) of AI/AN adolescents (ages 14–18 years) across key demographic characteristics.

We used descriptive statistics (e.g. frequency, percentages, modes, and crude rates per 100,000) to characterize the impact of suicidal thoughts, behaviors, and deaths for adolescent AI/ANs. Next, we computed the potential years of life lost at a lifespan of 80 years (YPLL80) using the CDC standard years of life lost before 80 years for AI/AN adolescents. The ages of suicidal deaths of the adolescents were subtracted from the standard years of life [24, 25]. We assumed virtually every U.S. resident should be able to reach the age of 80 years if they engage in healthy behaviors, have adequate social and financial resources, and have reasonable access to healthcare services [26]. The number of years representing differences between actual ages at death and 80 years of life was summed across all the years of study in each potential life span to obtain the total number of years of life lost due to adolescent AI/AN suicides for the potential life span. No ethical approvals were needed as we used publicly available deidentified data from the CDC website. Also, the CDC conducts its own ethical approval processes before collecting these data.

## Results

The YRBS had an aggregated sample of 590 AI/AN adolescents for the years 2021, 2019, 2017, and 2015 (Table 1). There were pronounced fluctuations in the percentage of adolescents who contemplated suicide (suicidal ideation) and they averaged approximately 1 in 4. In addition, the equivalent of 76% of those who contemplated suicide went on to plan suicide attempts, and the equivalent of 82% of those who planned suicide attempted suicide. A quarter of those who attempted suicide were injured to such an extent that they needed clinical care.

**Table 1:** Suicidal thoughts and behaviors among AI/AN adolescents

Year	*N*	Considered Attempting Suicide, %	Planned suicide, %	Attempted suicide, %	Injured due to suicide attempt, %
2021	145	27.3	21.9	16.0	1.0
2019	145	34.7	24.2	25.5	11.5
2017	137	19.0	13.7	6.8	2.4
2015	163	20.9	17.4	15.0	4.0
All years	590	25.8	19.7	16.2	4.1

Source: YRBS, CDC

During the four years of the study, a total of 257 AI/ AN adolescents died of suicide (Table 2). The majority of suicides were observed among males (62.5%). The crude suicide rates per 100,000 from 2015 to 2021 for males increased from 15.24 to 22.86 (50% increase) and for females, they increased from 13.52 to 14.88 (10% increase). Suicides among this population were more common in older adolescents, one-third of AI/AN adolescent suicides were observed among those 18 years of age. AI/ANs comprised 1.8% of the adolescent population in 2021. Thus, their total number of suicides per year was small compared to other racial/ethnic groups. However, their crude suicide death rate (18.95 per 100,000) was the highest of any racial/ethnic group (i.e. 70.4% greater than non-Hispanic Whites, 239% greater than non-Hispanic Blacks, 284.1% greater than Asians, and 298% greater than Hispanics) (Table 3). AI/AN adolescent suicides during the study period resulted in 15, 931 years of potential life lost before the age of 80 (Table 1). Throughout the study period, suffocation was used most often and averaged 57.5% of all suicides and firearms averaged 35.5% of all suicides. Less than a tenth (7%) of AI/AN adolescents used other suicide methods (e.g. poisoning, drowning, cutting, etc.) (Table 4).

**Table 2:** AI/AN adolescent suicides by age, gender, year

Age (years)	Suicides (*n*)	% of all suicides
14	23	9.2
15	43	17.1
16	46	18.3
17	56	22.3
18	83	33.1
**Year**	**Male**,* N***(%)**	**Female**,* N***(%)**
2021	40(22.86)	25(14.88)
2019	43(22.38)	27(14.46)
2017	45(23.56)	17(9.17)^*^
2015	29(15.24)	25(13.52)
**Year**	**Suicides (** *n* **)**	**YPLL(** *n* **)**
2021	65	4129
2019	70	4438
2017	62	3931
2015	54	3433

*the numbers less than 20 may be unreliable. Source: CDC WISQARS

**Table 3:** Adolescent suicide death rates by Race/Ethnicity in 2021

Race/Ethnicity	Crude rate (per 100,000)	Suicides (*N*)	Population (14–18 years)
AI/AN	18.95	65	342,920
Non-Hispanic White	10.43	1,159	11,111,144
Non-Hispanic Black	7.93	234	2,949,507
Asian	6.67	79	1,184,609
Hispanic	6.36	347	5,455,455

Source: CDC WISQARS

**Table 4:** AI/AN adolescent suicides by methods, 2015–2021

Year	Firearms *N* (%)	Suffocation (*n*, %)	Other (*n*, %)
**2021**	20 (30.8)	37(56.9)	8(12.4)
**2019**	26 (37.1)	43 (61.4)	1(1.4)
**2017**	23 (37.1)	36(58.1)	3 (4.8)
**2015**	20(37.0)	29(53.7)	5(9.3)

Source: CDC WISQARS

Other indicates = immolation, drowning, poisoning, cutting, etc

## Discussion

A multitude of insults, including historical and current, have impacted AI/ AN families’ health and mental well-being. We focused on one outcome of such insults, the suicidal thoughts and behaviors of AI/AN adolescents. A study using the 2006–2015 National Violent Death Reporting System of a mixed racial and ethnic group of adolescents ages 10–19 years found the leading methods used for suicide in AI/ANs were suffocation (58.3%), firearms (32.5%), and poisonings (3.8%) which are very similar to our findings for suffocation (56.9%) and firearms (30.8%) [27]. Our sample was smaller than the previous study, so we had to aggregate all “other methods” as the third leading method of suicide. However, poisonings were the largest component of the “other” group. Also, the previous study and our study found AI/AN adolescent males were more likely than females to die by suicide. Any differences in the results between our analysis and the previous study may be due to differences in the time frames of the studies and the age ranges utilized. As previously noted in our results, the methods used by AI/AN adolescents for suicide were similar to those used by U.S. adolescents in general [28, 29]. Most likely the common use of the methods is due to the widespread availability of these means and methods.

Large increases in suicide rates for non-Hispanic AI/ANs between 1999 and 2017, with the largest increases in AI/AN females (139%) and AI/AN males (71%) have helped draw attention to the potential risk factors for suicide in AI/AN tribes [30]. A variety of risk factors (antecedents) for suicidal thoughts and behaviors affecting AI/ANs in general, not just all adolescents, have been summarized as follows: being male, having depression, feelings of hopelessness, racial discrimination, alcohol and other drug abuse, poor school performance, family/friend history of suicides, physical/sexual abuse, low SES, family emotional neglect, being bullied, emotional/mental health issues (e.g. anxiety, ADHD, conduct disorder), feeling unsafe at school, exposure to gang activity, interpersonal conflict, being gay, sexually active, and adverse childhood experiences in general [31].

Adverse childhood experiences (ACEs) are harmful experiences through the age of 18 years. ACEs include emotional, physical or sexual abuse, physical neglect, witnessing intimate partner abuse, household substance abuse or mental illness, incarceration of family members, race-based discrimination, family divorces, and witnessing neighborhood violence are among the commonly assessed ACEs [32]. AI/ANs report the greatest number and variety of ACEs of any racial or ethnic group and female AI/ANs report higher average ACEs than AI/AN males [32, 33] Research has also found that youth with four or more ACEs are more likely than other races to be AI/AN (28.3%) [34]. This is especially relevant since ACEs have been associated with poor mental health outcomes, including higher perceived stress, higher odds of a major depressive disorder and generalized anxiety disorder, and greater substance use disorder [35]. Also, each additional ACE has been found to increase the odds of suicide attempts by 37%, depression (57%), PTSD symptoms (55%), and polydrug use (51%) [36].

The aforementioned ACEs are layered on top of a mental substrate of AI/AN historical intergenerational trauma (e.g. colonization and modern structural racism) [37]. The US government removed AI/ANs from ancestral lands, confined them to reservations, severely reduced their numbers through physical violence and starvation, increased serious infections (e.g. smallpox), forced children away from their families to distant boarding schools in attempts to “civilize savages” and eliminate their generative cultural assimilation. Such actions have negatively impacted tribes by reducing employment opportunities, and social connections, creating areas of concentrated poverty, establishing lower-quality schools, and increasing stressors and social problems, creating a social environment of limited resources [36]. AI/AN youth too often spend much of their lives in chronic adversity; the AI/AN child poverty rate is approximately 33% (about double the national rate of 16% in 2022) and the AI/AN rate has been above the national rate for decades [38]. A systematic review that examined socioeconomic status and youth mental health found low socioeconomic status youth were two to three times more likely to develop mental health problems than higher SES peers [39]. In addition, research indicates that adolescents who attend “high poverty schools” (e.g. more than 75% of students eligible for free or reduced-price meals) were twice as likely to have attempted suicide as adolescents from “low poverty schools” (25% or less of the students eligible for free or reduced-price meals) [40].

The impact of antecedents of suicide on mental health disorders begins in childhood and increases in frequency and severity during adolescence [41, 42]. Factors that impact the mental health of AI/AN adolescents include but are not limited to heredity, chronic adversity, poverty, racial discrimination, violence, access to quality mental health care, social isolation, and stigma related to mental health issues [31, 43]. The result is that AI/AN adolescents tend to have higher rates of substance abuse including alcohol, post-traumatic stress disorder, depression, anxiety, loneliness, violence, and suicide when compared to the general adolescent population [42, 43]. By the time of young adulthood (ages 18 to 25) serious mental illness is present in one in 14 AI/ANs [44]. One in eight adolescents ages 12 to 17 years had major depressive episodes, one in four used illicit drugs in the past year, one in 20 binged alcohol in the past month, and one in 30 had an alcohol use disorder in 2019 [43].

Interventions to reduce suicides among youths often attempt to do so by proactively identifying youths at increased risk for suicide ideation or suicide planning by questioning the youths and their friends regarding risk factors for suicide (secondary prevention). However, mental health professionals’ ability to predict if someone is going to commit suicide in the next year is no better than chance [45]. Because youths spend the majority portion of their time at school, personnel at schools are expected to recognize and refer at-risk students for appropriate help. A recent report found that there is no association between state suicide prevention training requirements of school personnel and youth suicide rates [46]. Attempts to predict suicide in adolescence may result in several potential harms including unnecessary referrals, student labeling, anxiety, possible stigma, and false positives and false negatives. Instead, the US Preventive Services Task Force has recommended screening for depression, a leading cause of suicide in adolescence [47].

Professionals who wish to reduce suicidal thoughts and behaviors (primary prevention) should focus more of their efforts on providing youths with protective factors also known as resilience factors to establish sufficient ego strength in them to adequately deal with all types of risk factors and stressors for potential suicidal thoughts and behaviors. A systematic review of resilience factors that moderate childhood adversity and the development of mental health problems for young people (ages 13 to 24) in general found 21 resilience factors [48]. The factors that moderated childhood adversity were: mental flexibility, cognitive reappraisal, lower rumination, high distress tolerance, lower aggression, low expressive suppression, low substance use expectancy, low insecure attachment, low rejection, low other-directedness, high self-esteem, low ego over and under control, high extended and immediate family support, family cohesion and positive family climate, parental involvement in youth activities, positive parenting behaviors, and high community level support. More specifically a systematic review of protective factors against suicidal thoughts and behaviors in AI/AN adolescents found the following protective factors: feeling positive about school, positive mood and self-image, subjective assessment and good physical health, high family connectedness, family cares about their feelings, high parental expectations, parents knowing where they have been when not at home, caring about child’s schoolwork, positive adult relationships in the community and with teachers, feeling safe at school and greater involvement with traditional cultural activities [49]. Both of the aforementioned reviews indicate how important the family is in moderating the effects of stressors that push youth towards suicidal thoughts and behaviors. A more recent study of AI/AN middle school students in grades 6–8 also found that high levels of family, community, and school support were important in mitigating suicidal thoughts, plans, and attempts [50]. Importantly these protective factors seem to be universal for adolescents, but the strength of specific factors in reducing the risk of suicide may vary by race and gender.

Other interventions for reducing AI/AN suicides include increasing mental health literacy, decreasing prejudice and discrimination, and starting early in the education of youths (e.g. comprehensive school health education). More specifically, to reduce firearm suicides, tribal leaders should pass laws requiring handguns to be locked away (means restriction) when not in use (e.g. child access prevention laws, CAP). Research has found that eight of the 10 top states with the highest firearm death rates are among the group of states without CAP laws [51, 52]. State-level inactions have also contributed to mental health problems for the AI/AN population in other ways (e.g. low income, high poverty, inadequate education facilities, lower access to utilities, poor mental health funding, just to name a few) [52, 53]. Indigenous community agencies need to establish family-directed home-visiting suicide prevention education programs for parents potentially based on the American Indian Lifeskills Development curriculum [54]. Members of federally recognized tribes are eligible to receive free healthcare from the Indian Health Service. However, the IHS has been chronically underfunded for decades [55]. The IHS in 2017 had per capita funds one-half the per capita funds of Medicaid, and per capita funds were less than funding for prisoners in federal prisons [56]. The result is unidentified, untreated, and undertreated mental health problems. Also, there is a need to increase the number of physicians, psychologists, and teachers who are well-versed in American Indian cultures. AI/AN psychologists comprise 0.13% of psychologists and 0.004% of psychiatrists [57–59]. The proposed methods of ameliorating suicides in AI/AN adolescents are but the tip of the iceberg of needed interventions. The solution to AI/AN suicides must be multifactorial including economic, educational, medical, social, and cultural interventions.

There are several potential limitations to our study [60]. First, the CDC YRBS surveyed only adolescents who attended schools, and the data were self-reported. This could result in underreporting of non-fatal suicide attempts due to self-reporting bias. Second, our study focused on adolescents 14 to 18 years of age; however, the YRBS included a small sample of 19-year-olds as well. Thus, the YRBS and WISQARS data vary slightly in age ranges. Third, the WISQARS database did not contain numerous demographic variables such as education level, economic status, and family structure, that could have assisted with a more granular assessment of AI/AN adolescent suicides. Fourth, potential misclassification of suicides based on intent, race, or ethnicity was possible. Such misclassification could result in an underestimation of suicides by intent or by race or ethnicity. Fifth, due to relatively small numbers of suicides, state-based differences in suicide fatalities could not be assessed. Finally, due to cultural heterogeneity among 574 federally recognized tribes care should be taken to attribute these suicidal thoughts and behaviors, especially across all tribes.

## Conclusions

Suicide is the second leading cause of death for high school-age AI/AN adolescents. We found the prevalence of considering, planning, and attempting suicide has increased in AI/AN adolescents. In the past 20 years, there has been minimal success in reducing suicides in adolescent populations. Interventions to ameliorate the prevalence of adolescent suicides have not been broad enough in their targets to address suicidal thoughts and behaviors in racial/ethnic minority youth. The magnitude and intensity of the resources applied to suicide reduction efforts are too anemic to adequately impact the risk factors for suicides across various groups of youth. Federal, state, and tribal leaders must do more to address the epidemic of suicides in adolescent populations with special emphasis on high-risk groups such as AI/AN youth.

## Data Availability

All data files are publicly available at- https://www.cdc.gov/injury/wisqars/index.html. https://www.cdc.gov/yrbs/index.html.
